# Building Bridges Post-Licensure Consultation: Strengthening the Geropsychology Workforce

**DOI:** 10.1093/geroni/igaf122.781

**Published:** 2025-12-31

**Authors:** Erin Emery-Tiburcio, Erin Kube, Ann Steffen, Suzann Ogland-Hand, Julia Kasl-Godley, Michelle Mlinac, Janet Yang, Andrew Heck

**Affiliations:** Rush University Medical Center, Chicago, Illinois, United States; VISN 19 Clinical Resource Hub, Salt Lake City, Utah, United States; University of Missouri-St. Louis, St. Louis, Missouri, United States; Ogland-Hand Consulting, LLC, Grand Rapids, Michigan, United States; The Wright Institute, Berkely, California, United States; VA Boston Healthcare System, Boston, Massachusetts, United States; Heritage Clinic, Pasadena, California, United States; GeroPartners, LLC, Midlothian, Virginia, United States

## Abstract

U.S. Census trends indicate significantly increased numbers of older adults and thus, a greater need for medical and mental health providers equipped to address older adult needs. Since the Building Bridges Conference in March 2021, the Building Bridges Post-Licensure (BBPL) group has been working to advance clinical practice with older adults. In 2022, we conducted a needs assessment of 80 post-licensure psychologists and identified a strong desire for gero-focused case consultation. We subsequently launched two six-month pilot consultation groups, occurring monthly for 90 minutes and led separately by a board certified geropsychologist. The groups consisted of didactic teaching, case presentation, and case discussion, focusing on foundational knowledge, assessment, and intervention geropsychology competencies. Nine licensed psychologists and one licensed clinical social worker completed consultation and eight of these participants completed the post-assessment. Most agreed consultation added value to their clinical practice (62% ‘strongly agree’; 38% ‘agree), was responsive to their professional needs (75% ‘strongly agree’; 25% ‘agree’), and 88% strongly agreed they received direction with difficult cases. Qualitative accounts of skills implemented were highlighted. This presentation will describe the BBPL’s efforts in developing these groups, including recruitment strategies, structure and format of monthly groups, and outcome measures. This approach may serve as a model for other disciplines interested in developing post-licensure training and educational opportunities for providers working with older adults.

